# Peripheral Nerve Injury Induces an Interferon-Responsive State Centered on Satellite Glial Cells in the DRG That Sustains Neuropathic Pain Through STAT1 and CXCL10 Signaling

**DOI:** 10.3390/brainsci16090921

**Published:** 2026-08-29

**Authors:** Wenchao Hu, Futai Wang, Ziyi Niu, Peiyang Liu, Zhicheng Tian, Ceng Luo, Rougang Xie

**Affiliations:** 1 Department of Neurobiology, School of Basic Medicine, Fourth Military Medical University, Xi’an 710032, China; 18734963121@163.com (W.H.); wang_futai@163.com (F.W.); nzy1323323@163.com (Z.N.); 17829592518@163.com (P.L.); zhichengt@163.com (Z.T.); 2 The Shaanxi Province Key Laboratory of Brain Function Analysis and Modulation, Xi’an 710032, China

**Keywords:** neuropathic pain, satellite glial cells, STAT1, CXCL10–CXCR3 axis, dorsal root ganglion

## Abstract

**Highlights:**

**What are the main findings?**

- SNI induces a distinct STAT1-associated IFN SGC population in the DRG, characterized by increased CXCL10 expression.
- This SGC inflammatory program is associated with sensory-neuron hyperexcitability and pain hypersensitivity. Local STAT1 inhibition or CXCR3 blockade alleviates pain.

**What are the implications of the main findings?**

- SGCs may actively shape the DRG inflammatory microenvironment and drive neuronal sensitization after nerve injury.
- The STAT1–CXCL10–CXCR3 axis is a potential local therapeutic target for neuropathic pain and injury-related functional impairment.

**Abstract:**

**Background:** Peripheral nerve injury drives persistent remodeling of the dorsal root ganglion (DRG) microenvironment, but the cellular states and intercellular signaling mechanisms that sustain neuropathic pain remain poorly understood. **Methods:** Here, we integrated single-cell transcriptomics with histological, behavioral, and electrophysiological approaches to identify an interferon-responsive state in satellite glial cells (SGCs) of the DRG following spared nerve injury (SNI). **Results:** This state was distinguished by STAT1-associated interferon-responsive genes’ activation and enhanced chemokine signaling, particularly involving CXCL10 and its receptor CXCR3. These molecular alterations were accompanied by remodeling of intercellular communication among SGCs, sensory neurons, and immune cells. Pharmacological inhibition of STAT1 or blockade of CXCR3 not only reduced sensory-neuron hyperexcitability but also attenuated pain hypersensitivity and improved deficits in hindlimb weight bearing and gait after SNI. **Conclusions:** Collectively, our findings indicate that interferon-responsive SGCs (IFN SGCs) contribute to the maintenance of neuropathic pain by regulating STAT1-dependent chemokine signaling in the DRG. Targeting this signaling pathway may provide a therapeutic strategy for persistent neuropathic pain.

## 1. Introduction

Neuropathic pain arises from a lesion or disease affecting the somatosensory nervous system and may endure well beyond the stabilization of the initial insult [1,2]. Peripheral nerve injury produces sustained spontaneous pain, hyperalgesia, and allodynia, which are commonly associated with increased excitability of primary sensory neurons [3]. However, neuron-intrinsic changes alone are unlikely to account for the long-term maintenance of pain hypersensitivity. Accumulating evidence indicates that nerve injury remodels the DRG, where sensory neurons interact with glial, immune, and vascular-associated cells [4,5]. How this local microenvironment is reorganized to maintain sensory sensitization remains incompletely understood.

SGCs constitute a major glial population in the DRG and closely envelop the somata of sensory neurons [6]. Under physiological conditions, they regulate extracellular ionic balance, neurotransmitter clearance, metabolic support, and the perisomatic milieu [7]. Following nerve injury, SGCs undergo molecular and structural remodeling and can release inflammatory mediators that modulate neuronal excitability and local immune activity [8]. Despite their strategic location at the interface between injured neurons and the DRG microenvironment, SGCs are frequently regarded as a broadly activated population [9]. This perspective, however, inadequately captures the diversity of injury-induced glial responses and insufficiently identifies which SGC states may contribute to the transition from an acute injury response to persistent pain [10].

Interferon-responsive transcriptional programs are increasingly recognized as key regulators of tissue inflammation and cellular stress [11,12]. STAT1 serves as a major transcriptional mediator of interferon signaling and can regulate immune-cell recruitment and neuronal activity by inducing chemokines [13]. Chemokines such as CCL2, CXCL10, and CCL5 have individually been implicated in neuroinflammatory pain states [14,15]. Despite the established role of SGC reactivity in neuropathic pain, the specific functional states that govern their inflammatory outputs, their precise communication axes with sensory neurons, and their causal contribution to pain maintenance have not been fully elucidated.

In the present study, to address these questions, we employed the SNI model [16] and integrated single-cell transcriptomics with histological, electrophysiological, and behavioral analyses. We first characterized SGC state diversity in the injured DRG and examined whether a distinct IFN SGC state emerges during the maintenance phase of neuropathic pain. We next investigated whether this state engages STAT1-dependent chemokine signaling to modulate communication among SGCs, sensory neurons, and immune cells. Finally, we inhibited STAT1 or blocked the CXCL10 receptor CXCR3 following the establishment of pain hypersensitivity to determine whether this signaling network contributes to pain maintenance. Through this integrative framework, we sought to bridge injury-induced SGC state transitions with the multicellular signaling logic that sustains neuropathic pain.

## 2. Materials and Methods

### 2.1. Animals and Models

All animal procedures were performed in accordance with institutional guidelines and approved by the Institutional Animal Care and Use Committee of the Fourth Military Medical University. Adult male C57BL/6J mice (8–12 weeks old) were obtained from the Laboratory Animal Center of FMMU. Mice were housed under a 12 h light/dark cycle with ad libitum access to food and water. Male mice were assigned to the experimental groups without a predetermined preference. No formal computer-generated randomization procedure or stratification based on body weight or baseline behavioral responses was used. Investigators were blinded to group allocation during behavioral data acquisition and subsequent data analysis.

Mice exhibiting predefined humane endpoints, including >20% body-weight loss, inability to eat or drink, severe lethargy, or persistent pain or distress, were euthanized by gradual-fill CO_2_ inhalation, with death subsequently confirmed by cervical dislocation.

SNI model was used to induce neuropathic pain. All surgical procedures were performed under aseptic conditions. Mice were anesthetized by inhalation of isoflurane, and anesthesia was maintained throughout the procedure. The animals were placed on a thermostatically controlled heating pad to maintain body temperature. After anesthesia was established, the left sciatic nerve was exposed at the mid-thigh level by bluntly separating the overlying muscles. The tibial and common peroneal nerves were tightly ligated proximally and transected distal to the ligation, whereas the sural nerve was left intact. Sham-operated mice underwent the same anesthesia and surgical exposure of the left sciatic nerve, but the nerve branches were neither ligated nor transected.

Behavioral testing was performed 14 days after SNI or sham surgery. None of the animals exhibited overt surgery-related motor deficits, such as limb paralysis. Mice that reached predefined humane endpoints, including more than 20% body-weight loss, inability to eat or drink, severe lethargy, or persistent and unrelieved severe distress, were euthanized using gradual-fill CO_2_ inhalation, followed by confirmation of death by cervical dislocation.

### 2.2. Single-Cell RNA-Sequencing Data Analysis

Publicly available scRNA-seq data from mouse DRGs were downloaded from GEO under accession number GSE155622 [17]. The dataset included samples annotated as the control and SNI 14d. According to the GEO record, five control and two SNI 14d biological replicates were available. However, individual animal IDs were not provided in the public metadata, preventing the assignment of cells to specific animals and precluding animal-level pseudobulk analysis. Accordingly, comparisons between the control and SNI 14d were interpreted as descriptive and exploratory, and individual cells were not treated as independent biological replicates.

The raw UMI count matrix was processed in R version 4.6 using Seurat version 5.0. Cells with fewer than 200 or more than 5000 detected genes, or with a mitochondrial gene proportion greater than 10%, were excluded. The data were normalized using Seurat’s LogNormalize method with a scale factor of 10,000. The 2000 most variable genes were identified using the VST method, followed by data scaling and PCA. PCs 1–20 were used for neighbor-graph construction, clustering, and UMAP visualization. Clustering was performed using Seurat’s FindNeighbors and FindClusters functions with a resolution of 0.4 and the Louvain algorithm. Cluster-marker genes were identified using the Wilcoxon rank-sum test with only.pos = TRUE, min.pct = 0.25, and logfc.threshold = 0.5.

Cell types were annotated according to canonical marker genes: neurons (Nefh, Tubb3, and Elavl3), SGCs (Fabp7, Apoe, and Kcnj10), Schwann cells (Mpz, Pmp22, and Ncmap), macrophages (Adgre1 and Cd68), T cells (Cd3d and Cd3e), B cells (Cd19 and Ms4a1), neutrophils (S100a8 and S100a9), endothelial cells (Pecam1 and Cdh5), fibroblasts (Dcn and Col1a1), and vascular smooth muscle cells (Acta2 and Myh11).

SGCs were extracted and reanalyzed using the same general workflow. PCs 1–15 were used for neighbor-graph construction, clustering, and UMAP visualization, with a clustering resolution of 0.4. An IFN-response module score was calculated for each SGC using Seurat’s AddModuleScore function based on nine interferon-stimulated genes (ISGs): Isg15, Mx1, Oas1a, Ifit1, Ifit3, Stat1, Irf7, Ifi44, and Ifih1. SGCs with scores above the 75th percentile of all SGCs were classified as IFN SGCs. Among the remaining cells, those with Gap43 expression above the median were classified as reactive SGCs, whereas all other cells were classified as homeostatic SGCs. The assigned states were examined using feature plots and violin plots of representative genes, including Isg15, Stat1, and Ccl2 for IFN SGCs and Gap43, Atf3, and S100a11 for reactive SGCs. These categories were interpreted as computationally defined transcriptional states rather than definitive lineage or functional classifications.

Neurons were extracted for further analysis. Module scores were calculated using Seurat’s AddModuleScore function for CGRP-related neurons (Calca), IB4-related neurons (Smr2 and Pou4f2), and NF200-related neurons (Nefh). Each neuron was assigned to the category with the highest module score. Cells for which the highest module score was below 0.1 were classified as unclassified. The assignments were evaluated using feature plots, violin plots, and UMAP visualization and were interpreted as transcriptomic classifications.

A differential expression analysis was performed using Seurat’s FindMarkers function and the Wilcoxon rank-sum test. For cluster-marker identification, the parameters were only.pos = TRUE, min.pct = 0.25, and logfc.threshold = 0.5. Exploratory comparisons between the control and SNI 14d were performed in SGCs and IFN SGCs. Genes with an adjusted *p* value < 0.05 and an absolute log2 fold change >0.5 were considered candidate differentially expressed genes at the cell level. Because individual animal IDs were unavailable, cell-level *p* values were not interpreted as animal-level confirmatory statistics. Group differences were evaluated primarily according to the direction and magnitude of expression changes. GSEA was performed using the fgsea package and mouse Hallmark gene sets from MSigDB. Genes were ranked according to their log2 fold changes, and enriched pathways were interpreted as exploratory and hypothesis-generating.

Type I- and type II-IFN-related module scores were calculated using Seurat’s AddModuleScore function. The type I-IFN module included Isg15, Mx1, Oas1a, Ifit1, Ifit3, Stat1, Irf7, Ifi44, and Ifih1. The type II-IFN-related module included Cxcl9, Cxcl10, Icam1, Ciita, Gbp2, Stat1, Gbp4, Gbp6, and Socs1. Module-score distributions were compared descriptively between the control and SNI 14d in SGCs and IFN SGCs.

Transcription-factor regulatory-network activity was inferred using an SCENIC-like workflow [18], in which GENIE3 was used to construct candidate regulatory networks and AUCell was used to evaluate regulon activity. The inferred regulatory activities were interpreted as computational predictions rather than direct evidence of transcription-factor binding or activation.

Cell–cell communication was analyzed using LIANA with the mouse consensus ligand–receptor resource (mouse consensus) [19], which contains 3989 ligand–receptor pairs. For each ligand–receptor pair, communication strength was estimated as the product of the mean log-normalized expression of the ligand in the sender population and the receptor in the receiver population. Overall communication scores were calculated by summing the scores across ligand–receptor pairs for each sender–receiver cell-type pair. Analyses were performed separately for the control and SNI 14d. Chord diagrams were used to visualize the predicted communication network, and bubble plots were used to display chemokine-associated interactions between IFN SGCs and neuronal or immune-cell populations. Interactions involving Ccl2, Cxcl10, and their corresponding receptors were summarized descriptively. These results represent transcriptome-based ligand–receptor predictions and do not directly demonstrate ligand secretion, protein-level binding, receptor activation, or causal intercellular signaling. Therefore, the results were described as predicted communication rather than experimentally validated signaling.

Plots were generated using ggplot2 and Seurat-associated visualization functions.

### 2.3. qPCR

L3–L5 DRGs were collected from the left side of nine mice in each group. The DRGs from every three mice were pooled into one sample, resulting in three pooled biological samples per group. Total RNA was extracted from each pooled sample using TRIzol reagent (Invitrogen, Carlsbad, CA, USA), and the RNA concentration and purity were assessed using a NanoDrop spectrophotometer. Equal amounts of RNA from each pooled sample were treated for genomic DNA removal and reverse-transcribed using the Evo M-MLV RT Kit with gDNA Clean for qPCR II (Cat# AG11728, Accurate Biology, Changsha, China), generating three original cDNA samples per group.

Quantitative real-time PCR was performed using SYBR^®^ Green Pro Taq HS Premix II (Cat# AG11740, Accurate Biology, Changsha, China) on a Bio-Rad CFX96 Touch™ Real-Time PCR Detection System (Table 1). Each cDNA sample was analyzed in three technical replicates in each of three independent qPCR experiments. For each independent experiment, the three technical replicates were averaged to obtain one mean value for the corresponding pooled cDNA sample. Thus, three pooled cDNA samples were each measured in three independent qPCR experiments, generating nine assay-level measurements per group. The three independent qPCR experiments represented technical-level repetitions and were not considered additional biological replicates.

Relative target-gene expression was normalized to Gapdh and calculated using the 2^−ΔΔCt^ method. The amplification protocol consisted of an initial denaturation at 95 °C for 30 s, followed by 40 cycles of 95 °C for 5 s and 60 °C for 30 s. Melt-curve analysis was performed after amplification to confirm the specificity of the products. Comparisons between the sham and SNI groups were performed using an unpaired two-tailed Student’s *t*-test.

### 2.4. Immunofluorescence Labeling and Confocal Analysis

Male C57BL/6 mice were used, with 6 animals in total, including 3 in the sham group and 3 in the SNI 14-day group (*n* = 3/group). The mice were anesthetized by intraperitoneal injection of 1% sodium pentobarbital and transcardially perfused sequentially with PBS and 4% PFA. The ipsilateral L5 DRGs were dissected and post-fixed in 4% PFA for 3 h and then dehydrated in 30% sucrose/PB solution until the tissue sank. Dehydrated DRGs were embedded in OCT compound and sectioned at 10 μm using a cryostat. Sections were washed with PBS for 10 min and incubated with blocking solution (PBS containing 5% bovine serum albumin BSA and 0.3% Triton X-100) for 1 h at room temperature. Primary antibodies were then applied as follows: anti-CXCR3 antibody (rabbit, Sangon Biotech, Shanghai, China, 1:200) and anti-NeuN antibody (chicken, GeneTex, Irvine, CA, USA, 1:200) for co-staining; and anti-CXCL10 antibody (mouse, R&D Systems, Minneapolis, MN, USA, 1:200) and anti-glutamine synthetase (GS) antibody (rabbit, GeneTex, Irvine, CA, USA, 1:200) for co-staining. The sections were incubated overnight at 4 °C. After they were washed three times with PBS for 10 min each, corresponding secondary antibodies were applied: Alexa Fluor 488-conjugated donkey anti-rabbit IgG (Jackson ImmunoResearch, West Grove, PA, USA, 1:800), Alexa Fluor 594-conjugated donkey anti-chicken IgG (Jackson ImmunoResearch, West Grove, PA, USA, 1:800), Alexa Fluor 488-conjugated donkey anti-mouse IgG (Jackson ImmunoResearch, West Grove, PA, USA, 1:800), and Alexa Fluor 594-conjugated donkey anti-rabbit IgG (Jackson ImmunoResearch, West Grove, PA, USA, 1:800). The sections were incubated in the dark for 2 h at room temperature, washed three times with PBS for 10 min each, and then stained with DAPI (Sigma-Aldrich, St. Louis, MO, USA, 1:2000) for 10 min at room temperature in the dark. After three additional washes with PBS for 10 min each, fluorescence images were acquired using an Olympus FV3000 confocal microscope (Olympus, Tokyo, Japan). ImageJ 1.53t software was used for cell counting and fluorescence analysis. CXCR3-positive and CXCL10-positive cells and co-localized cells were manually counted, and the distribution characteristics of co-labeled neurons were analyzed. All counting experiments were performed by an investigator blinded to the experimental groups. Cell contours were manually outlined using the measurement tools in Olympus cellSens 4.5 software, and the equivalent circular diameters of double-positive cell profiles were measured in two-dimensional tissue sections. The measured diameters were grouped into predefined bins, and the relative frequency of cells within each diameter bin was calculated. The resulting diameter–relative-frequency distributions were fitted independently with Gaussian curves for the sham and SNI groups using the Gaussian fitting function in GraphPad Prism.

### 2.5. Local DRG Administration

Because the SNI procedure was performed on the left side, drugs were locally administered to the left L4 and L5 DRGs. Fludarabine (Flu) was prepared in artificial cerebrospinal fluid (aCSF) at a final concentration of 5 μg/μL and administered at 10 μL per DRG, corresponding to a dose of 50 μg per DRG. AMG487 was prepared in aCSF at a final concentration of 100 μM and administered at 10 μL per DRG, corresponding to a dose of 1 nmol per DRG. Because both the left L4 and L5 DRGs were injected, the total dose per animal was 100 μg of fludarabine or 2 nmol of AMG487. The control animals received an equivalent volume of the corresponding vehicle.

Animals were anesthetized with inhaled isoflurane. After adequate anesthesia was established, the animals were placed in the prone position with the left surgical side facing the operator. The skin over the iliac-crest region was shaved and disinfected using standard aseptic procedures. Under sterile conditions, the needle of a 1 mL syringe was bent by approximately 20° and loaded with 10 μL of the drug solution. The needle was inserted near the midline of the iliac crest, passed through the skin and muscle, and slowly advanced beneath the transverse process toward the intervertebral foramen. A slight loss of resistance was used to identify the target intervertebral foramen, and the DRG at the corresponding iliac-crest level was identified as the L5 DRG.

After localization, the superficial membranous tissue overlying the L5 DRG was gently pierced with the bent needle, and the corresponding drug solution was slowly delivered into the local region surrounding the L5 DRG to establish local drug exposure. The needle was left in place briefly after injection to minimize reflux and was then withdrawn slowly. The needle was subsequently advanced rostrally along the iliac crest, and the left L4 DRG was localized and injected using the same procedure. After completion of the L4 and L5 DRG injections, the animals were monitored postoperatively.

Following drug administration, the animals were allowed to acclimatize to the experimental environment for 1 h before behavioral testing or acute isolation of the corresponding DRGs for whole-DRG patch-clamp recording. Animals were evaluated according to prespecified exclusion criteria. Animals showing apparent damage to the dura mater or spinal cord surface, persistent abnormal behavior, severe motor impairment, obvious limb paralysis, or any other condition likely to affect the reliability of behavioral or electrophysiological measurements were excluded from subsequent analyses.

The dura mater and spinal cord were not further incised or disrupted during the procedure. The purpose of this approach was to establish a local drug-exposure environment around the target DRGs rather than to perform intrathecal or intraspinal administration.

### 2.6. Ex Vivo Whole-Cell Patch-Clamp Recording of DRG Neurons

Ex vivo whole-cell patch-clamp recordings were performed to assess the electrophysiological activity of mouse DRG neurons. After euthanasia, the left L4/L5 DRGs ipsilateral to the SNI were rapidly exposed, dissected, and transferred to a dish containing aCSF. After the removal of the surrounding connective tissue, the intact DRGs were incubated in an enzyme solution containing trypsin (0.4 mg/mL) and type I collagenase (1.0 mg/mL; Sigma, St. Louis, MO, USA) at 37 °C for 10 min. This enzymatic treatment was used to mildly digest the membranous tissue on the DRG surface, thereby exposing the underlying neurons and facilitating patch-clamp sealing. After the removal of the superficial membranous tissue, the DRGs were transferred to aCSF continuously bubbled with a mixture of 95% O_2_ and 5% CO_2_ and allowed to recover at 28 °C for at least 1 h before recording. The aCSF contained the following (in mM): 125 NaCl, 2.5 KCl, 1.2 NaH_2_PO_4_, 1.0 MgCl_2_, 2.0 CaCl_2_, 26 NaHCO_3_, and 10 of glucose.

During recording, neurons within the intact DRGs were visualized using an upright microscope equipped with infrared differential interference contrast optics and a 40× water-immersion objective (BX51WI, Olympus, Tokyo, Japan). Whole-cell patch-clamp recordings were performed using an EPC-10 amplifier and PatchMaster v2x90.3 software. The resistance of the recording electrodes after filling with the internal solution was 5–8 MΩ. Small- to medium-diameter neurons with somatic diameters of less than 30 μm were selected for recording.

In current-clamp mode, the membrane potential was adjusted to approximately −70 mV before current injection, and evoked action potentials were recorded. The pipette internal solution contained the following (in mM): 126 potassium gluconate, 10 NaCl, 1 MgCl_2_, 10 EGTA, 2 Na-ATP, and 0.1 Mg-GTP. The pH was adjusted to 7.4 with KOH, and the osmolarity was approximately 300 mOsm.

Cells were excluded from subsequent analysis if a high-resistance seal could not be established or maintained, the leak current exceeded 100 pA, the membrane properties were unstable, or the recording quality was otherwise inadequate. The DRG tissue used for each animal group was obtained from three animals. In the statistical graphs, the sample size (n) represents the number of successfully recorded cells included in the analysis, and each data point represents one recorded cell.

### 2.7. Behavioral Testing

All behavioral experiments were conducted during the light phase between 09:00 and 17:00 in a quiet room with relatively stable lighting and temperature. Before formal testing, mice were habituated to the behavioral testing environment for 3 consecutive days for 30–60 min per day. On each testing day, mice were allowed to acclimate to the corresponding apparatus for at least 1 h before assessment. Investigators conducting behavioral tests were blinded to group allocation and drug-treatment information.

For all behavioral assays, the individual mouse was considered the experimental unit. Repeated trials within the same mouse were averaged to obtain one value for that animal before statistical analysis. Therefore, the reported sample size (n) represents the number of animals, rather than the number of stimulus applications or repeated trials. The exact number of animals in each group is provided in the corresponding figure legends.

### 2.8. Mechanical Hypersensitivity Assessment (Von Frey Test)

Mechanical responses were assessed using von Frey filaments. Before testing, mice were individually placed in black compartments on an elevated metal mesh platform and allowed to habituate for at least 1 h. Von Frey filaments with the following forces were used: 0.008, 0.02, 0.04, 0.07, 0.16, 0.40, 0.60, 1.0, 1.4, and 2.0 g (Danmic Global, Campbell, CA, USA).

In the SNI model, filaments were applied to the lateral plantar surface of the ipsilateral hind paw. Testing began with the lowest-force filament. Each filament was applied perpendicularly to the plantar surface until slight bending occurred and was maintained for approximately 2 s. Paw withdrawal, paw lifting, or paw licking was recorded as a positive response. Each filament was applied 10 times, and the number of positive responses was recorded. Mechanical hypersensitivity was expressed as the number of positive responses/10. These response proportions were then fitted using the Cumulative Gaussian–Percents function in GraphPad Prism to calculate the 50% withdrawal threshold.

### 2.9. Thermal Hypersensitivity Assessment (Hargreaves Test)

Thermal hypersensitivity was assessed using a Hargreaves apparatus (IITC model 400, Woodland Hills, CA, USA). Mice were placed on a glass platform maintained at 30 °C. Following habituation, a focused radiant heat source positioned beneath the glass was applied to the plantar surface of the hind paw. The time from onset of thermal stimulation to paw withdrawal was recorded as the paw withdrawal thermal latency (PWTL).

Each hind paw was tested four times with an interval of at least 5 min between trials, and the mean latency was used as the thermal nociceptive threshold for that paw. To prevent tissue injury, the cutoff time was set at 25 s. If no withdrawal response occurred within 25 s, stimulation was terminated, and the latency was recorded as 25 s.

### 2.10. Brush Test

The brush test was used to assess dynamic mechanical allodynia. A camel-hair brush approximately 2 mm in width was gently brushed across the ipsilateral plantar surface from heel to toes for approximately 2 s. Responses were scored according to the intensity of defensive behavior:

Score 0: No obvious response.

Score 1: Mild or transient paw withdrawal followed by rapid recovery.

Score 2: Clear paw withdrawal accompanied by brief paw licking or shaking.

Score 3: Strong avoidance behavior, including sustained paw withdrawal, repeated paw licking or biting, escape behavior, or whole-body guarding posture.

The interval between stimulations was at least 15 s. Each mouse was tested repeatedly, and the mean score was used to evaluate dynamic mechanical sensitivity.

### 2.11. Cold Plate Test

Cold sensitivity and cold hypersensitivity were assessed using a cold plate apparatus (BIO-CHP, BIOSEB, Vitrolles, France). Before testing, the metal plate was set and maintained at 4 ± 0.5 °C. The surface temperature was calibrated using an external thermocouple thermometer to ensure stable testing conditions.

On the test day, the mice were habituated in the experimental room for at least 30 min. The baseline responses were assessed 1–2 days before surgery, and tests were performed at designated time points after surgery. During testing, the mice were gently placed in the center of the cold plate and confined within a transparent acrylic cylinder. Timing began immediately after the mice contacted the cold plate. The ipsilateral hind paw was observed for pain-like behaviors, including paw lifting, shaking, rapid withdrawal, or licking. Timing was stopped, and latency was recorded upon the first occurrence of any of these behaviors.

The latency to the first pain-like behavior was used as the cold nociceptive threshold. To avoid tissue injury or excessive stress caused by prolonged cold exposure, a cutoff time of 60 s was applied. If no obvious response occurred within 60 s, the latency was recorded as 60 s. Each mouse was tested three times at each time point, with an interval of at least 15–20 min between trials, and the mean of the three trials was used for statistical analysis.

### 2.12. CatWalk

The CatWalk XT gait analysis system (Noldus Information Technology, Wageningen, The Netherlands) was used to assess gait and affected-limb function in the mice. Before formal testing, the mice underwent 3 days of habituation training. During training, the mice were placed at one end of the runway and allowed to traverse the glass walkway voluntarily. Training was conducted three times per day to ensure that the mice could complete continuous, stable runs.

For formal testing, the mice freely traversed a 20 × 10 cm glass walkway. Paw-print signals were recorded by a high-speed camera positioned beneath the glass. The CatWalk 10.6.608 software automatically identified paw contacts with the glass surface and calculated parameters, including paw contact duration, contact intensity, contact area, walking speed, and step sequence, to evaluate changes in locomotor function and affected-limb use after SNI.

To ensure data stability, the camera gain was set to 20 dB, and the detection threshold was set to 0.1. At each time point, each mouse was required to complete three compliant runs. A compliant run was defined as one with a run duration between 0.5 and 20 s and a maximum speed variation of no more than 60%. The mean of the three compliant runs was used for statistical analysis.

### 2.13. Quantification and Statistical Analysis

Group allocation was known to the investigators responsible for animal assignment and experimental treatment. Investigators performing outcome assessment and data analysis were blinded to group allocation using coded identifiers until completion of the analysis. Statistical analyses were performed using GraphPad Prism 8.0. Normality and homogeneity of variance were assessed using the Shapiro–Wilk and Levene’s tests, respectively. Normally distributed data were analyzed using two-tailed paired or unpaired *t*-tests, one-way ANOVA, or repeated-measures ANOVA, as appropriate. Non-parametric tests were used when parametric assumptions were not met. Data are presented as means ± SEMs, with *p* < 0.05 being considered statistically significant.

## 3. Results

### 3.1. Single-Cell Analysis of the DRG After SNI Reveals Reactive Remodeling and Expansion of an IFN-Associated Inflammatory State in Satellite Glial Cells

**Figure 1:**
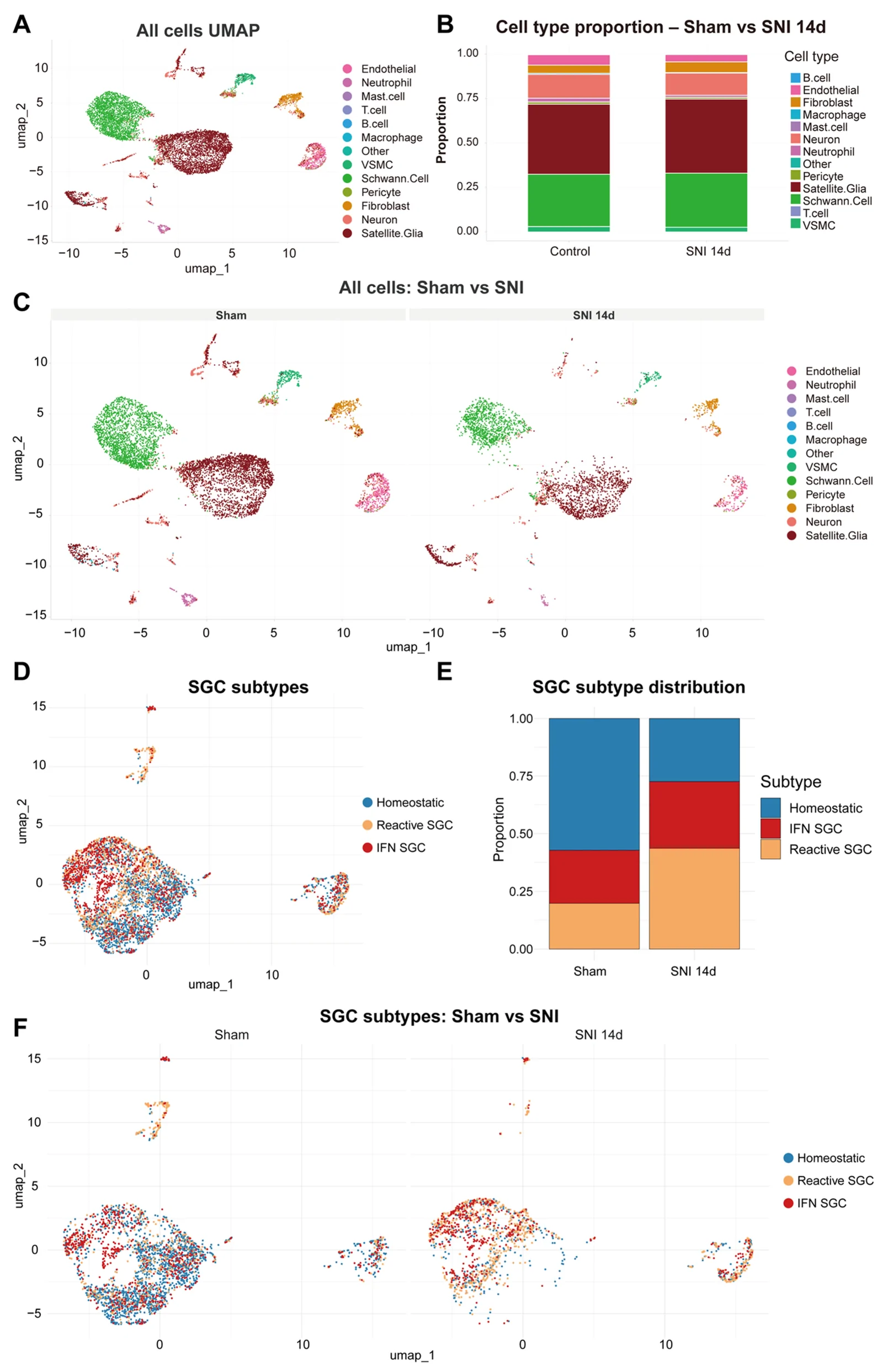
Single-cell profiling reveals reactive remodeling and identifies an interferon-responsive state in satellite glial cells after SNI. (**A**) UMAP visualization of the integrated single-cell RNA-sequencing dataset, showing the major cell populations identified in the DRG. (**B**) Relative representation of major cell populations among captured cells from sham-operated mice and mice at day 14 after SNI. (**C**) Integrated UMAP embedding shown separately for the sham and SNI day 14 groups. (**D**) UMAP visualization of reclustered SGCs, identifying homeostatic, reactive, and interferon-responsive transcriptional states. (**E**) Relative representation of the three SGC states in the sham and SNI day 14 groups. (**F**) SGC UMAP embedding shown separately for the sham and SNI day 14 groups.

**Figure A1:**
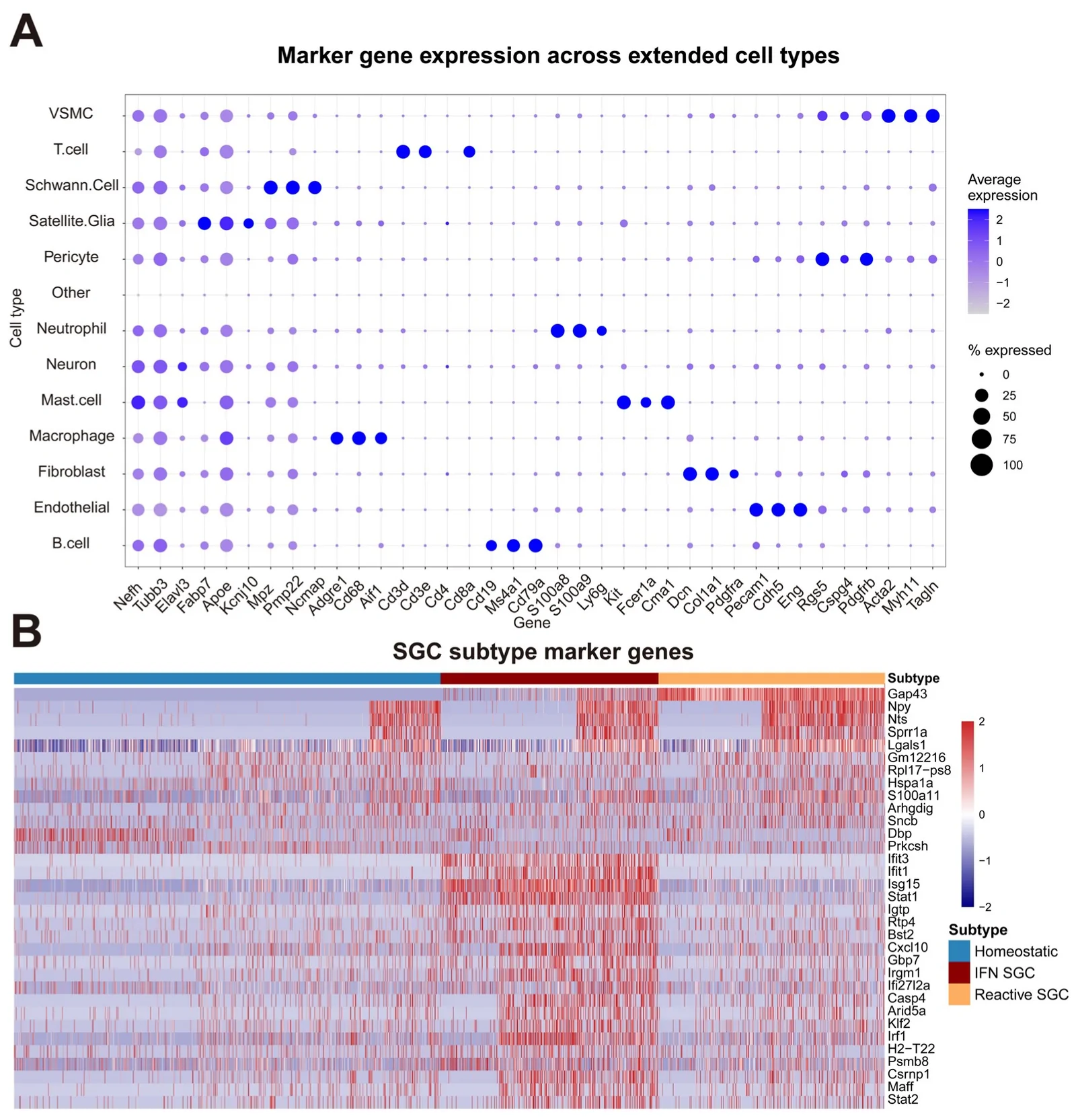
Cell-type annotation and SGC subtype identification in the DRG. (**A**) Dot plot showing the expression of canonical marker genes across major DRG cell types. Dot size indicates the percentage of expressing cells, and color indicates average expression level. (**B**) Heatmap showing the top 20 marker genes of homeostatic SGCs, IFN SGCs, and reactive SGCs.

To systematically characterize changes in the cellular composition of the DRG microenvironment and its potential pathological remodeling after peripheral nerve injury, we integrated and analyzed single-cell RNA-sequencing datasets from DRGs harvested from sham-operated mice and mice 14 days after SNI [17]. SNI is a well-established model of neuropathic pain induced by peripheral nerve injury, which induces persistent mechanical hypersensitivity, enhanced sensory-neuron excitability, and reactive alterations in glial and immune cells within the DRG, and thus it suitable for resolving the long-term remodeling of the post-injury DRG microenvironment [16]. After quality control and data integration, we generated a single-cell atlas of the major cellular populations in the DRG (Figure A1A). Cell identities were assigned according to canonical lineage markers and cluster-enriched genes. UMAP revealed multiple transcriptionally distinct and spatially separated cell clusters (Figure 1A). This cellular organization was consistent with the known composition of the DRG, in which neuronal and non-neuronal cells collectively form a complex peripheral sensory microenvironment.

We next compared the overall cellular composition of DRGs between the sham and SNI day 14 groups. Cells from both groups occupied the same major cellular compartments in the integrated UMAP, indicating that SNI did not lead to the complete loss of any major cell population (Figure 1C). Neurons, SGCs, and Schwann cells remained the predominant populations in both conditions, with SGCs forming a prominent and well-defined cluster. The overall proportion of SGCs remained comparable between groups, whereas the distribution of SGC states underwent substantial changes after SNI (Figure 1B,C). In addition, several non-neuronal populations, including immune and vascular/stromal populations, exhibited changes in their relative representation after injury. These findings indicate that SNI-associated remodeling of the DRG microenvironment involves coordinated changes across neuronal, glial, immune, vascular, and stromal compartments rather than a neuron-restricted response.

In the global atlas, SGCs were highly abundant and displayed prominent injury-associated changes in their cellular states. Given their capacity to shift from homeostatic support cells to reactive glia and to modulate neuronal injury signaling, we focused subsequent analyses on the SGC compartment. SGCs were first identified on the basis of their canonical marker profile and subsequently classified into three mutually exclusive states through a hierarchical strategy. The IFN-response module score was calculated with Seurat’s AddModuleScore function based on the average expression of nine interferon-stimulated genes: Isg15, Mx1, Oas1a, Ifit1, Ifit3, Stat1, Irf7, Ifi44, and Ifih1. SGCs with an IFN-response module score above the 75th percentile of all SGCs, corresponding to the highest-scoring 25% of cells, were defined as IFN SGCs. Among the remaining SGCs, cells with Gap43 expression above the median level of all SGCs were defined as reactive SGCs, whereas the remaining cells were classified as homeostatic SGCs.

Subclustering revealed three distinct SGC states. In addition to the previously reported homeostatic SGCs [20], and reactive SGCs [21], we identified the remaining SGC cluster on the basis of its distinct transcriptomic features and defined it as the IFN-responsive SGC state, which was clearly distinct from the homeostatic and reactive populations (Figure 1D–F). A heatmap of SGC-subcluster marker genes supported this classification and revealed distinct transcriptional profiles across the three states. Homeostatic SGCs were enriched for genes associated with glial homeostasis, lipid metabolism, ion homeostasis, and intercellular communication, consistent with a basal supportive state that maintains the perisomatic microenvironment of sensory neurons. Reactive SGCs were characterized by programs associated with injury responses, extracellular matrix remodeling, inflammatory regulation, and tissue repair. By contrast, IFN SGCs exhibited prominent expression of canonical ISGs and chemokine-associated genes, indicative of an inflammatory or antiviral-like transcriptional state (Figure A1B). These findings demonstrate that SGCs do not undergo uniform activation after SNI; rather, they diversify across multiple injury-associated states characterized by attenuation of homeostatic programs, enhanced reactive remodeling, and activation of an interferon-associated inflammatory program.

### 3.2. SNI Preferentially Amplifies STAT1/ISG Activity in IFN SGCs

**Figure 2:**
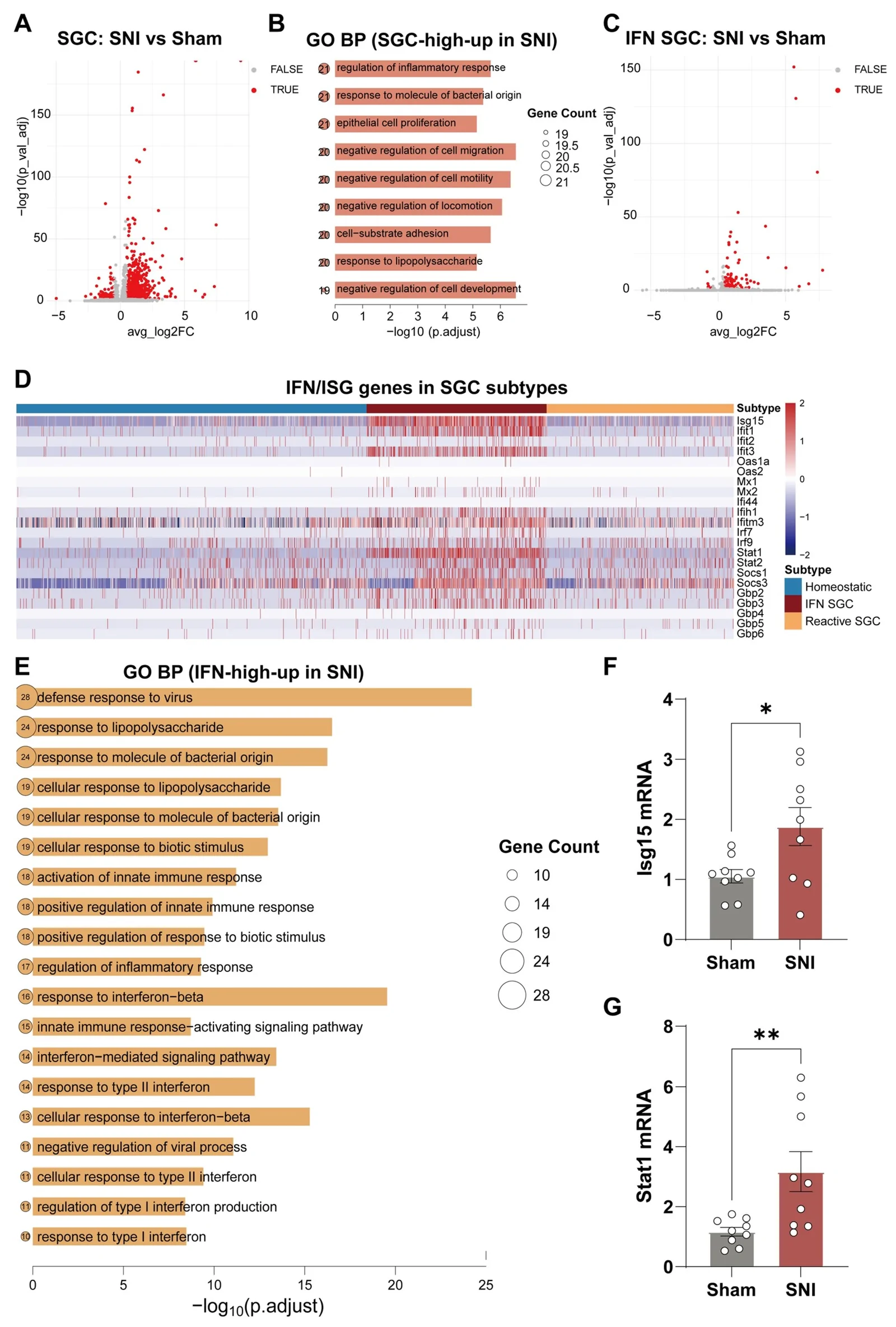
SNI induces inflammatory transcriptional remodeling and amplifies a STAT1/ISG program in satellite glial cells. (**A**) Volcano plot showing differentially expressed genes in SGCs between the SNI and Sham groups. (**B**) Gene ontology biological process (GO BP) enrichment analysis of genes upregulated in SGCs after SNI. Numbers and bubble sizes indicate GO-term gene counts; the x-axis shows −log10(p.adjust). (**C**) Volcano plot showing differentially expressed genes in IFN SGCs between the SNI and Sham groups. (**D**) Heatmap showing the expression of IFN/ISG-related genes across SGC subtypes. (**E**) GO BP enrichment analysis of genes upregulated in IFN SGCs after SNI. Numbers and bubble sizes indicate GO-term gene counts; the x-axis shows −log10(p.adjust). (**F**,**G**) Quantitative PCR analysis of Stat1 and Isg15 mRNA expression levels in the DRGs of the sham and SNI groups, *n* = 3. Statistical analysis was performed using an unpaired two-tailed Student’s *t*-test. Data are presented as means ± SEMs; * *p* < 0.05, ** *p* < 0.01.

To define injury-associated transcriptional remodeling in SGCs, we compared the overall SGC population between sham and SNI day 14 samples. Genes upregulated after SNI were enriched in inflammatory-response regulation, cell adhesion, migration, and tissue-remodeling processes (Figure 2A,B), indicating that SGCs develop coordinated inflammatory and structural-response programs following nerve injury.

IFN SGCs were next examined to determine whether their transcriptional state was altered by SNI. A prominent injury-associated response was identified in this population through differential expression analysis (Figure 2C). Across SGC states, IFN SGCs showed the strongest expression of STAT/IRF regulators and canonical ISGs, including Stat1, Irf7, Irf9, Isg15, Ifit family members, Oas family members, and Gbp genes (Figure 2D) [22]. These genes were expressed at lower or more heterogeneous levels in homeostatic and reactive SGCs, defining IFN SGCs as a distinct STAT1/ISG-high state.

**Figure A2:**
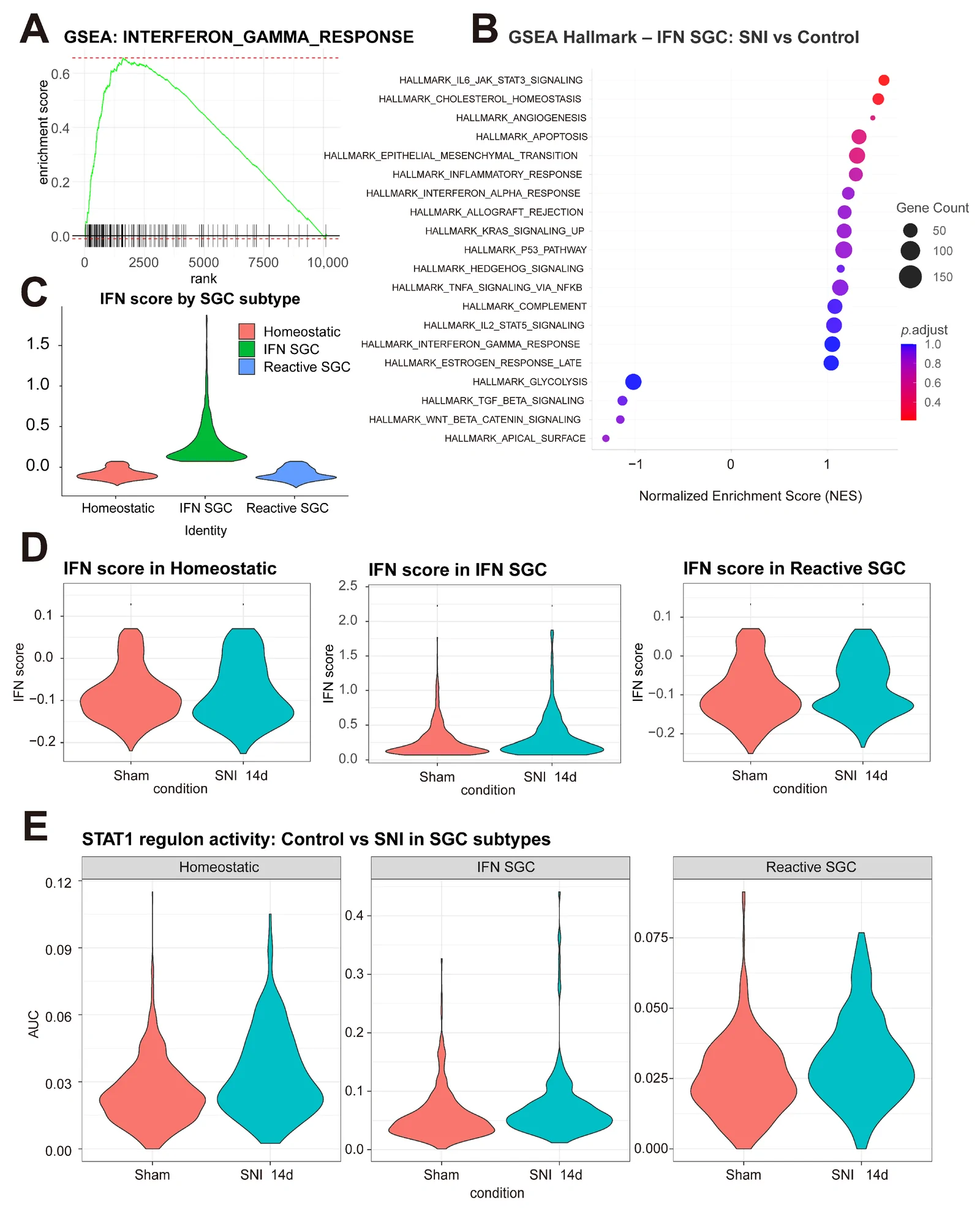
Enhanced interferon-responsive signaling and STAT1 regulon activity in SGCs after SNI. (**A**) GSEA enrichment plot for the INTERFERON_GAMMA_RESPONSE gene set. (**B**) GSEA of Hallmark pathways in IFN SGCs from SNI versus control DRGs. Dot size indicates gene count, and color indicates adjusted *p* value. (**C**) IFN scores across SGC subtypes. −0.0784 ± 0.0022 for homeostatic SGCs, 0.3056 ± 0.0114 for IFN SGCs, and −0.0710 ± 0.0032 for reactive SGCs. (**D**) Comparison of IFN scores in homeostatic, IFN-responsive, and reactive SGCs between control and SNI groups. For homeostatic SGCs, the values were −0.0786 ± 0.0024 (Sham) and −0.0770 ± 0.0058 (SNI 14d); for IFN SGCs, the values were 0.2822 ± 0.0119 and 0.3444 ± 0.0229; and for Reactive SGCs, −0.0769 ± 0.0049 and −0.0662 ± 0.0043, respectively. (**E**) Comparison of STAT1 regulon activity, assessed by AUCell scores, among SGC subtypes between control and SNI groups. Sham and SNI 14d were 0.0249 ± 0.0005 and 0.0329 ± 0.0014 in homeostatic SGCs, 0.2822 ± 0.0119 and 0.3444 ± 0.0229 in IFN SGCs, and 0.0256 ± 0.0009 and 0.0320 ± 0.0009 in reactive SGCs, respectively. Data are presented as means ± SEMs.

Genes induced by SNI in IFN SGCs were enriched for type I and type II interferon responses [23], cytokine-mediated signaling, and innate immune activation (Figure 2E) [24]. Consistently, GSEA showed enrichment of interferon-response programs along with IL6–JAK–STAT3, TNF–NF-κB, and complement-related pathways (Figure A2A,B) [25]. Virus-response GO terms were driven by canonical ISGs and reflected endogenous interferon signaling rather than evidence of viral infection.

IFN signature scores were highest in IFN SGCs and were further increased after SNI (Figure A2C,D). Increased IFN scores were also observed in reactive SGCs after injury, although their activity remained lower than that of IFN SGCs. A similar pattern was exhibited by STAT1 regulon activity: SNI increased STAT1 activity across SGC states, but both baseline activity and injury-associated activation were greatest in IFN SGCs (Figure A2E). Thus, SNI induces a broader STAT1-associated glial response while preferentially amplifying this program in IFN SGCs.

**Figure A3:**
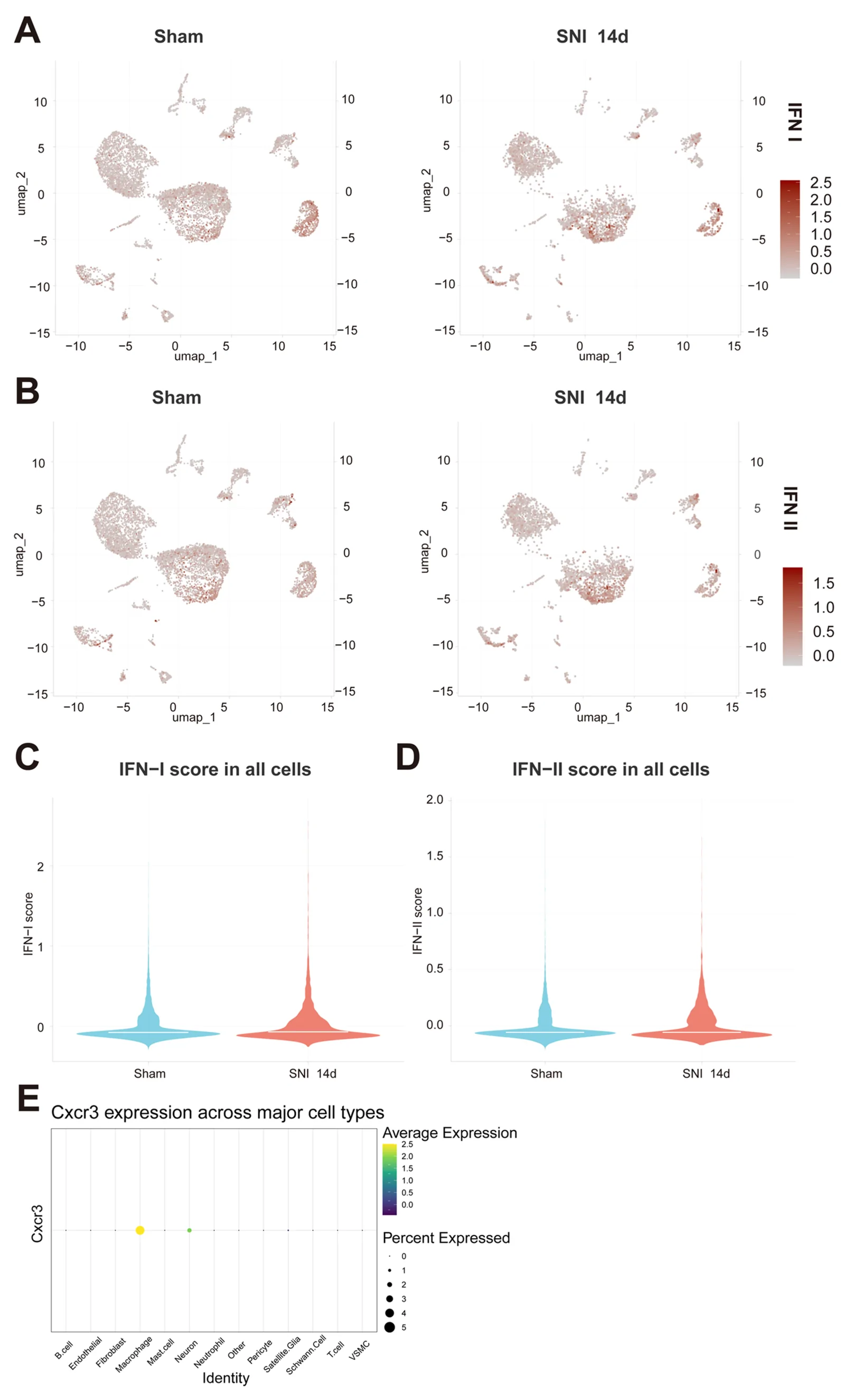
Global interferon-response signatures in the DRG after SNI. (**A**) UMAP feature plots showing IFN-I response scores in control and SNI DRGs. (**B**) UMAP feature plots showing IFN-II response scores in control and SNI DRGs. (**C**) Violin plots showing the distribution of global IFN-I response scores in all cells from the sham (−0.0195 ± 0.0056) and SNI 14d (−0.0451 ± 0.0107) groups. (**D**) Violin plots showing the distribution of global IFN-II response scores in all cells from the sham (0.0221 ± 0.0065) and SNI 14d (0.0381 ± 0.0109) groups. (**E**) Dot plot showing Cxcr3 expression across major cell types in the DRG. Data are presented as means ± SEMs.

At the tissue level, increased *Stat1* and *Isg15* expression in the DRG 14 days after SNI was confirmed by qPCR (Figure 2F,G) [26]. Type I and type II IFN signatures were also elevated across the injured DRG, particularly in non-neuronal regions (Figure A3A–D). Together, these findings show that a pre-existing IFN SGC state is amplified by SNI and identify this population as a major cellular carrier of the STAT1/ISG program in the injured DRG.

### 3.3. SNI Couples IFN SGCs to Chemokine-Mediated Neuron, Glia, and Immune Signaling

**Figure 3:**
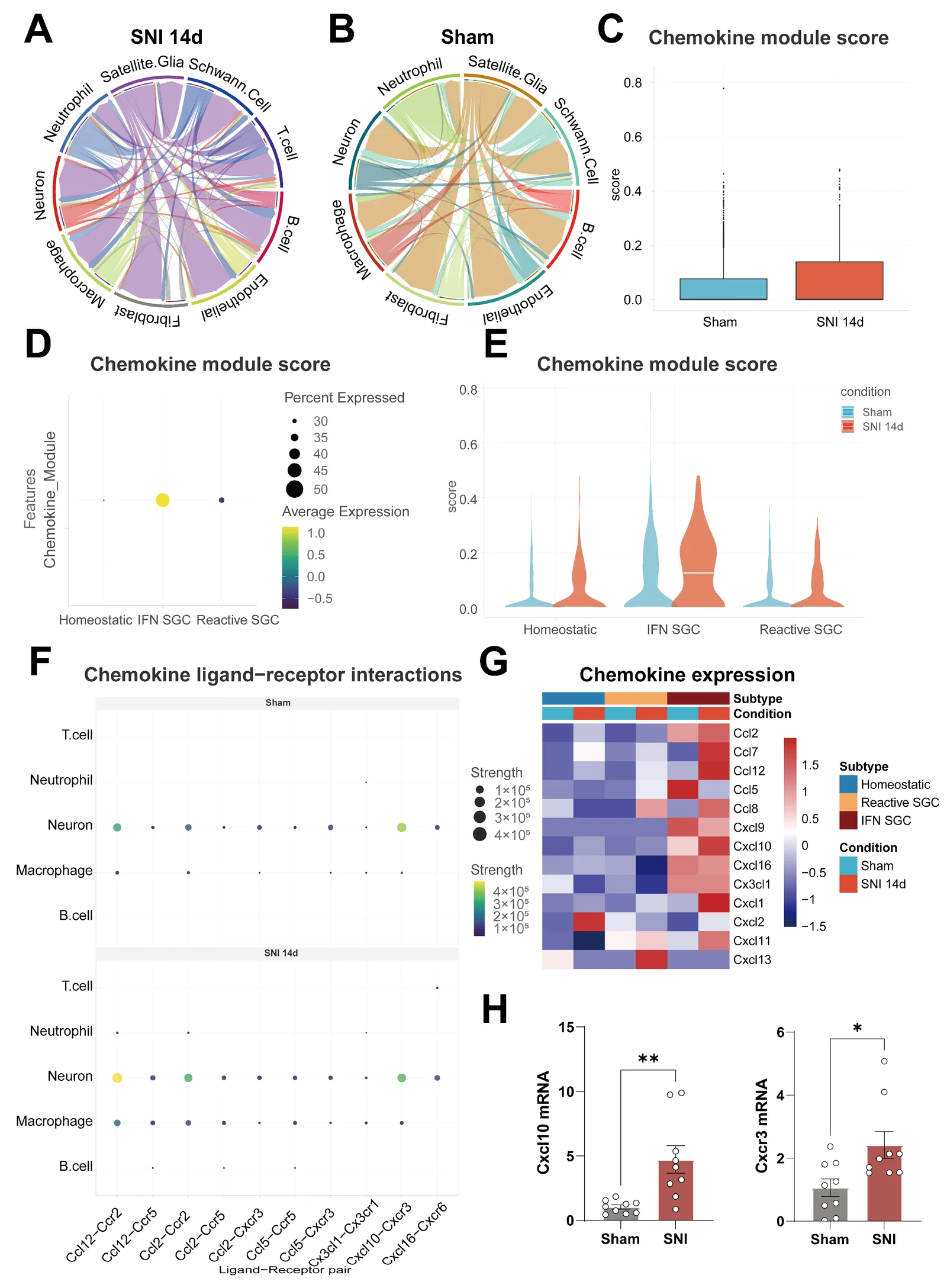
Exhibit enhanced chemokine communication with neurons and immune cells in the DRG after SNI. (**A**,**B**) Chord diagrams showing predicted intercellular communication networks among major cell types in the DRGs of the Sham group and the group at day 14 after SNI. (**C**) Chemokine module scores in all SGCs from the Sham group (0.0475 ± 0.0022) and the group at day 14 after SNI (0.0798 ± 0.0039). (**D**) Dot plot showing chemokine module activity across homeostatic, IFN-responsive, and reactive SGC subtypes. Dot size indicates the percentage of cells expressing genes in the chemokine module, and color indicates the average expression level. (**E**) Comparison of chemokine module scores across SGC subtypes between the Sham group and the group at day 14 after SNI. Sham and SNI 14d groups were 0.0320 ± 0.0022 and 0.0574 ± 0.0065 for Homeostatic SGCs, 0.0925 ± 0.0064 and 0.1354 ± 0.0087 for IFN SGCs, and 0.0417 ± 0.0048 and 0.0560 ± 0.0045 for Reactive SGCs, respectively. (**F**) Predicted interactions between chemokine ligands expressed by SGCs and receptors expressed by neurons and immune cells in the Sham group and the group at day 14 after SNI. Dot size and color indicate the predicted interaction strength. (**G**) Heatmap showing chemokine ligand expression across SGC states and experimental conditions. (**H**) Quantitative PCR analysis of Cxcl10 and Cxcr3 mRNA expression in the DRGs of the Sham and SNI groups. *n* = 3, data are presented as the mean ± SEM. * *p* < 0.05, ** *p* < 0.01.

Global ligand–receptor analysis revealed a dense intercellular communication network in the sham DRG, involving neurons, SGCs, Schwann cells, endothelial cells, fibroblasts, and immune populations (Figure 3B). At 14 days after SNI, the overall organization of this network was markedly redistributed, with altered interaction patterns observed among neuronal, glial, vascular, stromal, and immune compartments (Figure 3A). Notably, SGCs maintained extensive connections with multiple cell populations, including neurons, Schwann cells, endothelial cells, and immune cells. These results indicate that the intercellular signaling landscape of the DRG is reorganized by SNI, with SGCs constituting a major component of the injury-associated communication network.

The altered communication network was accompanied by state-dependent activation of a chemokine program within SGCs. Chemokine module activity was increased across the SGC population following SNI and was highest in IFN SGCs, where it was further enhanced after injury (Figure 3C–E) [27]. Reactive SGCs showed a smaller increase, whereas homeostatic SGCs retained comparatively low activity. Consistent with this pattern, the IFN-inducible chemokines Cxcl9, Cxcl10, and Cxcl11 were preferentially enriched in IFN SGCs, whereas broader or state-dependent expression was exhibited by other CCL and CXCL family members (Figure 3G). These findings connect the STAT1/ISG-high state to an injury-amplified chemokine output program.

Ligand–receptor inference further delineated this chemokine program into two putative signaling routes: SGC-to-neuron signaling and SGC-to-immune-cell signaling involving macrophages, neutrophils, and T cells (Figure 3F). These predicted interactions suggest that sensory neuronal states might be influenced by SGC-derived chemokines, while immune-cell recruitment or activation within the injured DRG might also be regulated. Consistent with these predictions, chemokine expression was broadly elevated in reactive and IFN SGCs after SNI, with prominent changes in the CCL2–CCR2 and CXCL10–CXCR3 signaling axes (Figure 3F,G). At the tissue level, qPCR analysis of DRGs collected 14 days after SNI further confirmed significant increases in Cxcl10 and Cxcr3 expression compared with sham controls (Figure 3H), supporting activation of the CXCL10–CXCR3 axis during the established phase of nerve injury. The cellular distribution of Cxcr3 across the major DRG populations was also examined using the single-cell transcriptomic dataset. Cxcr3 expression was most prominent in macrophages, whereas lower but detectable expression was also observed in neurons (Figure A3E). It was suggested by this distribution that CXCL10 may act on both immune and neuronal compartments and provided a rationale for further validating the neuronal localization of CXCR3 at the protein level.

**Figure 4:**
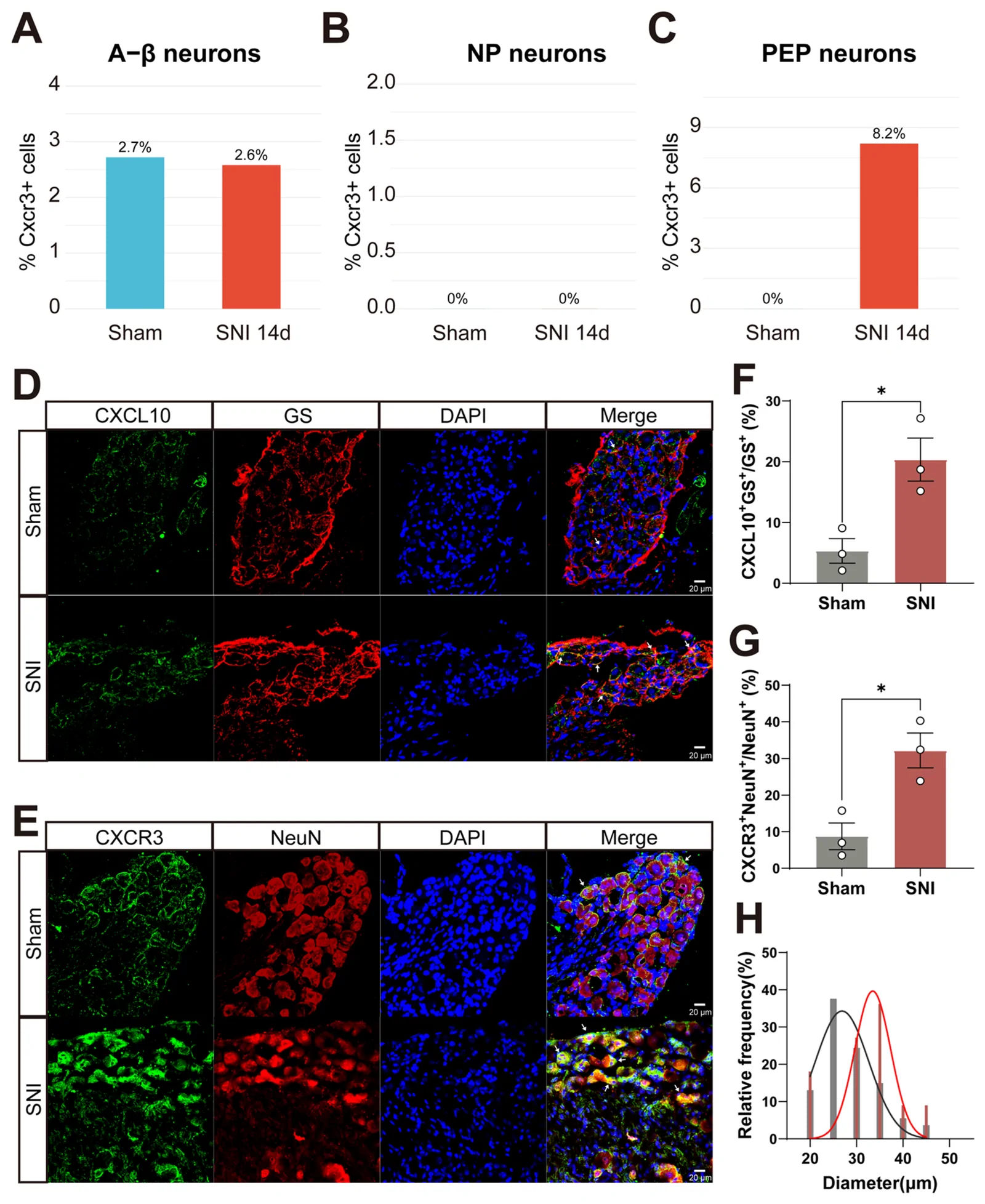
Cell-specific expression and localization of CXCL10 and CXCR3 in mouse L5 dorsal root ganglia at day 14 after SNI. (**A**–**C**) Percentages of CXCR3-positive cells among Aβ, peptidergic (PEP), and non-peptidergic (NP) neuronal subpopulations in the Sham and SNI 14d groups. (**D**) Representative confocal images showing CXCL10 (green), the satellite glial cell (SGC) marker GS (red), and DAPI (blue) in DRG sections. (**E**) Representative confocal images showing CXCR3 (green), the neuronal marker NeuN (red), and DAPI (blue) in DRG sections. Scale bar, 20 μm. (**F**) Quantification of CXCL10 and GS colocalization, expressed as the percentage of CXCL10^+^GS^+^ cells among total GS^+^ cells. (**G**) Quantification of CXCR3 and NeuN colocalization, expressed as the percentage of CXCR3^+^NeuN^+^ cells among total NeuN^+^ cells. (**H**) Relative-frequency distributions and Gaussian fits of DRG cell diameters in the Sham and SNI 14d groups. Data are presented as the mean ± SEM; *n* = 3 mice per group. At least three randomly selected nonconsecutive sections per mouse were analyzed under blinded conditions. Statistical comparisons in (**F**,**G**) were performed using an unpaired two-tailed Student’s *t*-test. * *p* < 0.05.

To determine whether the CXCL10–CXCR3 axis identified by transcriptomic and ligand–receptor analyses was localized to specific cellular compartments within the DRG, the distribution of CXCL10 and CXCR3 was next examined by immunofluorescence 14 days after SNI. CXCL10 was detected in satellite glial cells, as indicated by its colocalization with GS, and the proportion of CXCL10^+^GS^+^/GS^+^ SGCs was significantly increased after SNI compared with sham controls (Figure 4D,F). In parallel, CXCR3 immunoreactivity was observed in NeuN^+^ sensory neurons and was markedly enhanced after SNI, with the proportion of CXCR3^+^NeuN^+^/NeuN^+^ neurons increasing significantly compared with sham animals (Figure 4E,G). Analysis across sensory neuronal subtypes revealed that CXCR3 expression was detected in a small proportion of Aβ neurons in both the sham and SNI groups, whereas it was absent from NP neurons. Notably, CXCR3 expression was selectively induced in peptidergic (PEP) neurons after SNI, reaching 8.2% compared with 0% in sham controls (Figure 4A–C). The distribution of CXCR3^+^ neuronal diameters was also shifted toward larger-diameter neurons after SNI (Figure 4H). Together, these findings provide cellular-level validation of the injury-associated CXCL10–CXCR3 signaling axis, showing that SNI enhances CXCL10 production in SGCs and induces CXCR3 expression in a distinct subset of sensory neurons, particularly PEP neurons.

### 3.4. Pharmacological Suppression of STAT1-Associated Signaling and Blockade of CXCR3 Attenuate SNI-Induced Hypersensitivity and Neuronal Hyperexcitability

**Figure 5:**
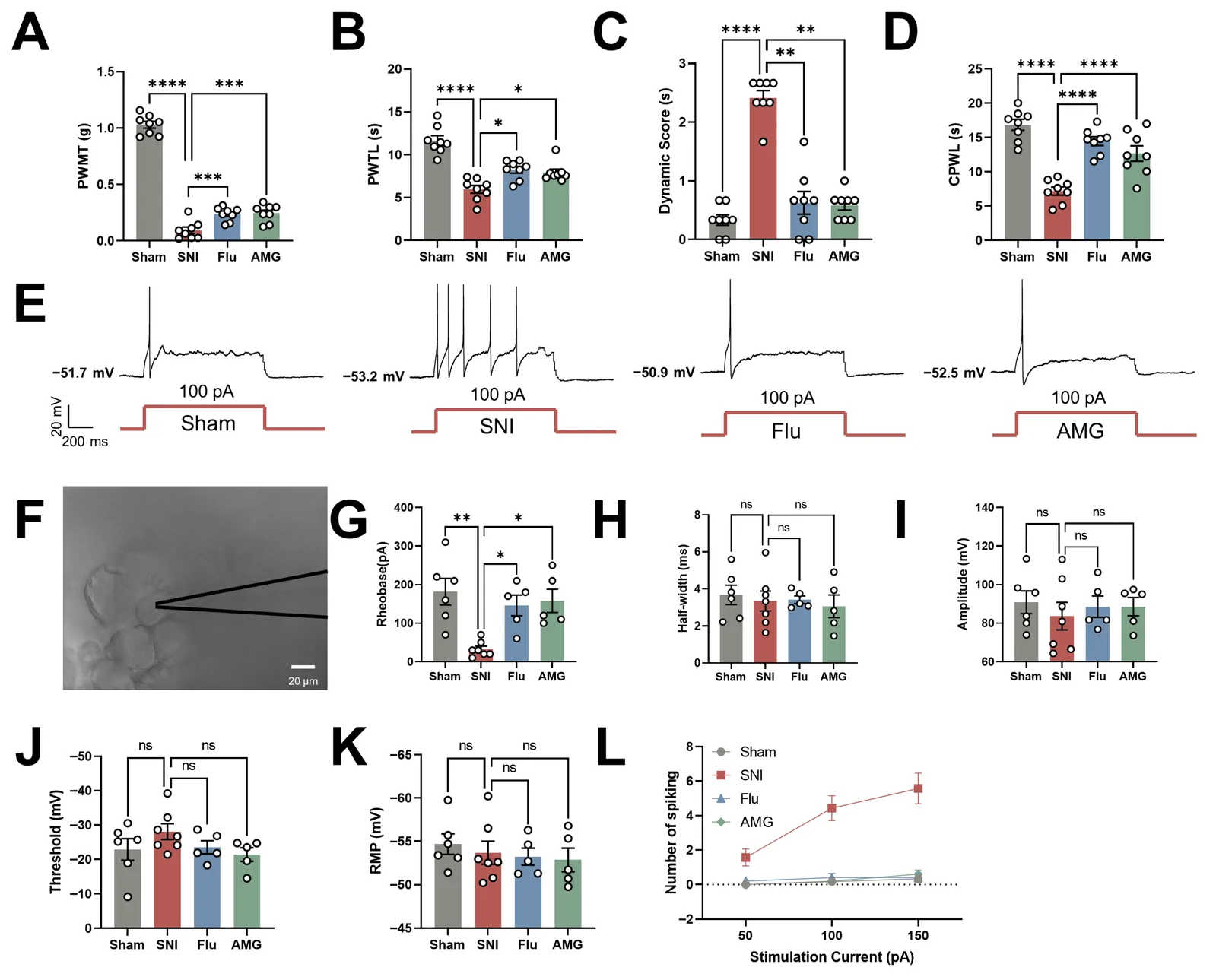
Fludarabine and AMG487 alleviate pain-related behaviors and reduce DRG neuron hyperexcitability after SNI. (**A**–**D**) Assessment of mechanical, thermal, dynamic mechanical, and cold pain behaviors in the sham, SNI, flu, and AMG groups. (**E**) Representative action potential traces of DRG neurons from each group. *n* = 8. (**F**) Representative image of patch-clamp recording from a DRG neuron. (**G**–**K**) Analyses of action potential rheobase, half-width, amplitude, threshold, and resting membrane potential. (**L**) Number of action potentials elicited by different current injections. *n* = 3. Statistical comparisons were performed using one-way analyses across the four groups. Depending on the distributional characteristics of the data and the homogeneity of variance, an ordinary one-way ANOVA, followed by Tukey’s multiple-comparisons test, Welch’s one-way ANOVA, followed by Dunnett’s T3 multiple-comparisons test, or the Kruskal–Wallis test, followed by Dunn’s multiple-comparisons test, was used, as appropriate. Data are presented as means ± SEMs; ns, not significant; * *p* < 0.05, ** *p* < 0.01, *** *p* < 0.001, **** *p* < 0.0001.

To functionally validate the STAT1/ISG and chemokine signaling networks identified by single-cell analysis, two predicted signaling levels were pharmacologically targeted: STAT1-associated inflammatory signaling with fludarabine [28] and chemokine receptor signaling with the CXCR3 antagonist AMG487 [29]. Mice were randomly assigned to the sham or SNI group before surgery. On postoperative day 14, 10 μL of aCSF, fludarabine, or AMG487 at the indicated concentration was directly injected into the DRG via a minimally invasive procedure. aCSF was administered to sham and vehicle-treated SNI mice, whereas fludarabine or AMG487 was given to drug-treated SNI mice. Behavioral tests were performed 1 h after the corresponding injection. At the day 14 baseline assessment, multimodal sensory hypersensitivity was induced by SNI, characterized by a reduced mechanical withdrawal threshold, shortened heat- and cold-response latencies, and increased dynamic mechanical allodynia scores. Both pharmacological interventions significantly attenuated these behavioral abnormalities across mechanical [30], and cold modalities [31] (Figure 5A–D). The convergent effects of targeting an upstream inflammatory program and a downstream chemokine receptor validate the functional relevance of the predicted signaling network in SNI-induced hypersensitivity.

The convergence of both interventions on the neuronal hyperexcitability phenotype was further demonstrated by whole-cell patch-clamp recordings. DRG neurons from SNI mice exhibited a reduced rheobase and generated more action potentials in response to graded current injections than neurons from sham mice (Figure 5E–G,L). Fludarabine and AMG487 attenuated the reduction in rheobase and suppressed excessive repetitive firing. In contrast, action potential half-width, amplitude, voltage threshold, and resting membrane potential remained unchanged across groups (Figure 5H–K). Thus, the treatment-associated reduction in excitability was characterized by the selective normalization of firing evoked by current injection rather than detectable changes in resting membrane potential or action potential waveform.

The transcriptomically defined inflammatory network was functionally validated at the neuronal level after SNI by the parallel attenuation of multimodal hypersensitivity and DRG neuronal hyperexcitability with fludarabine and AMG487. These convergent pharmacological effects implicate STAT1-associated and CXCR3-dependent signaling in injury-induced neuronal hyperexcitability and position CXCR3 as a pharmacologically targetable effector in the reorganized DRG signaling network. Together with the preferential expression of Cxcl10 in interferon-responsive SGCs, these data point to the CXCL10 and CXCR3 axis as a functional link connecting the injury-induced SGC chemokine program to persistent sensory dysfunction.

### 3.5. Targeting STAT1-Associated Signaling or CXCR3 Improves Dynamic Ipsilateral Hind Paw Use After SNI

**Figure 6:**
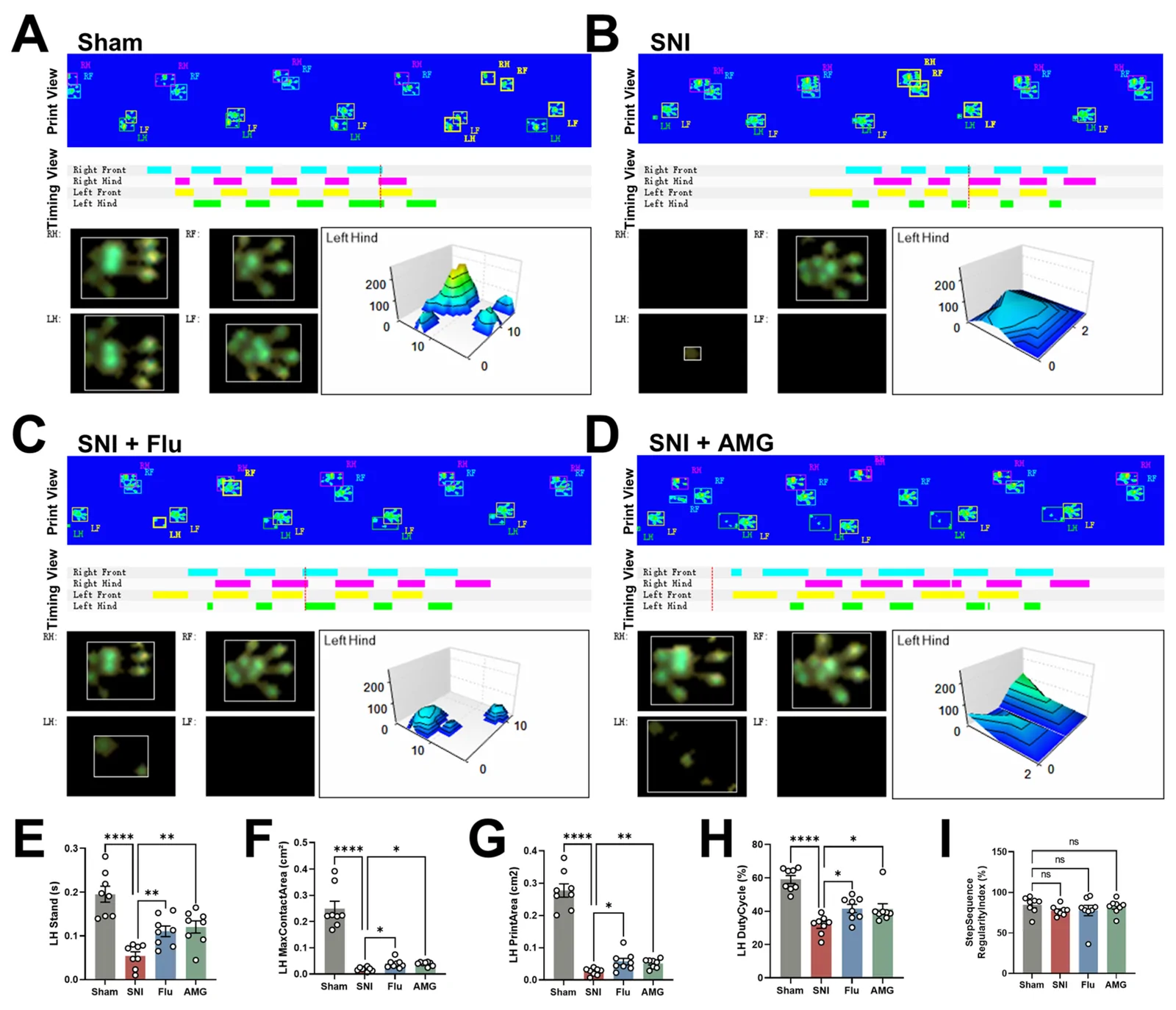
Fludarabine and AMG487 improve gait abnormalities and plantar pressure distribution following SNI. (**A**) Representative footprint patterns, gait timing, paw contact-intensity maps, and three-dimensional pressure distribution maps of the left hind paw in the sham group. (**B**) Representative footprint patterns, gait timing, paw contact-intensity maps, and three-dimensional pressure distribution maps of the left hind paw in the SNI group. (**C**) Representative footprint patterns, gait timing, paw contact-intensity maps, and three-dimensional pressure distribution maps of the left hind paw in the SNI + Flu group. (**D**) Representative footprint patterns, gait timing, paw contact-intensity maps, and three-dimensional pressure distribution maps of the left hind paw in the SNI + AMG group. (**E**) Quantitative analysis of left hind paw stand duration in each group. (**F**) Quantitative analysis of the maximum contact area of the left hind paw in each group. (**G**) Quantitative analysis of the left hind paw-print area in each group. (**H**) Quantitative analysis of the left hind paw duty cycle in each group. (**I**) Quantitative analysis of step sequence regularity in each group; *n* = 8. Statistical comparisons were performed using one-way analyses across the four groups. Depending on the distributional characteristics of the data and the homogeneity of variance, an ordinary one-way ANOVA, followed by Tukey’s multiple-comparisons test, Welch’s one-way ANOVA, followed by Dunnett’s T3 multiple-comparisons test, or the Kruskal–Wallis test, followed by Dunn’s multiple-comparisons test, was used, as appropriate. Data are presented as means ± SEMs; ns, not significant; * *p* < 0.05, ** *p* < 0.01, **** *p* < 0.0001.

Whereas the preceding assays measured stimulus-evoked hypersensitivity, the CatWalk [32] analysis was employed to determine whether the effects of fludarabine and AMG487 extended to dynamic use of the affected limb during locomotion. Representative footprint and contact-intensity maps showed a pronounced loss of ipsilateral hind paw contact after SNI, with smaller paw prints and diminished plantar contact signals relative to sham controls (Figure 6A,B). Both treatments increased the detectable ipsilateral paw-print signal and partially restored the plantar contact pattern compared with untreated SNI mice (Figure 6C,D).

The quantitative analysis resolved this phenotype into complementary temporal and spatial components. SNI reduced both stand duration and duty cycle of the ipsilateral hind paw, indicating decreased participation of the affected limb in the support phase of the gait cycle (Figure 6E,H). In parallel, print area and maximum contact area were markedly reduced, demonstrating a spatial contraction of plantar contact during stepping (Figure 6F,G). Fludarabine and AMG487 significantly improved all four measures, shifting both the duration and area of ipsilateral paw contact toward sham levels. Thus, a coordinated improvement across distinct dimensions of affected-limb use was produced by the two interventions, rather than an alteration of a single gait parameter.

**Figure A4:**
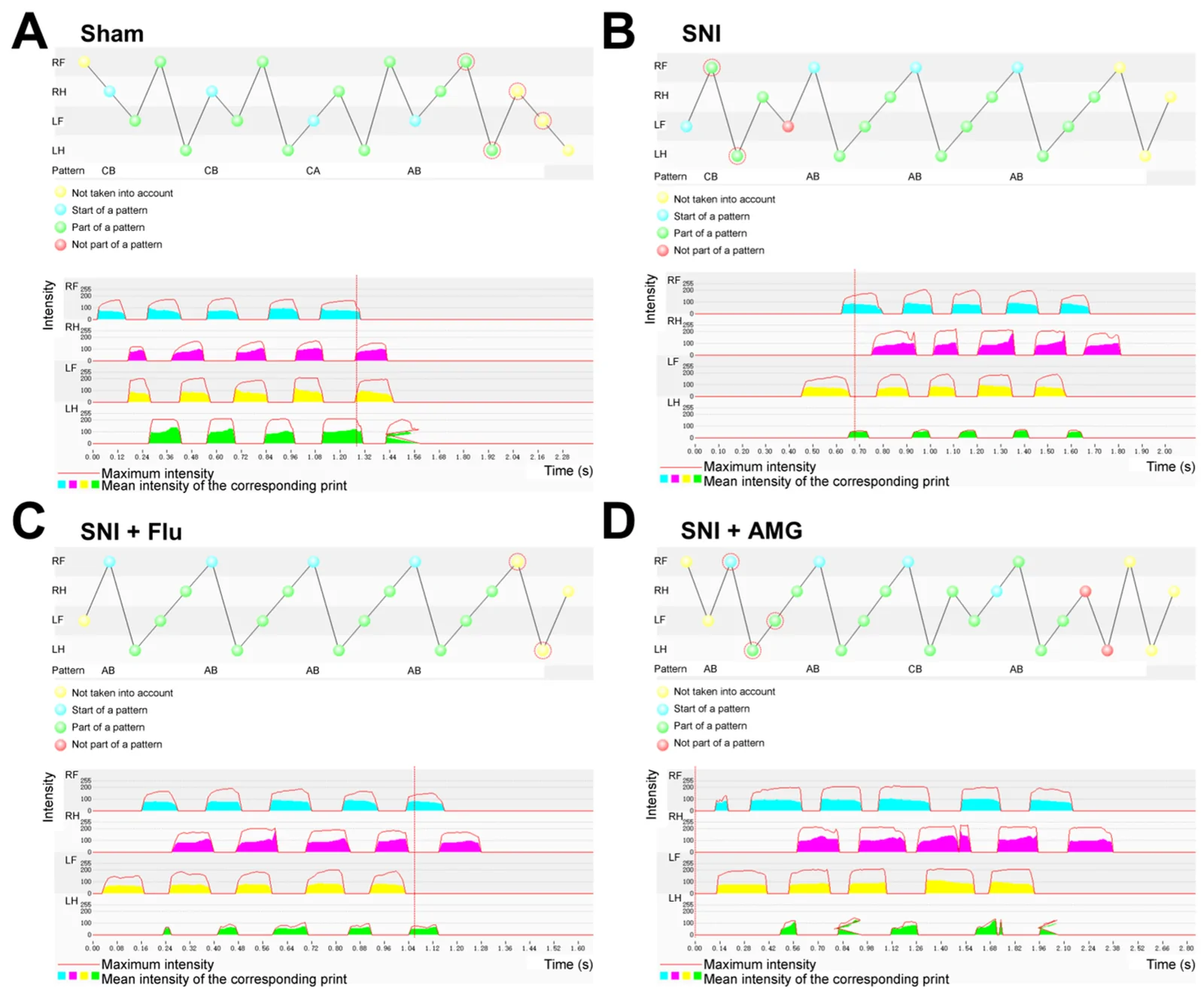
Fludarabine and AMG487 improve gait timing and interlimb coordination after SNI. (**A**–**D**) Representative gait sequence diagrams and paw pressure-versus-time curves in the sham (**A**), SNI (**B**), SNI + Flu (**C**), and SNI + AMG (**D**) groups. The upper panels depict interlimb step sequence and coordination, whereas the lower panels show contact intensity and temporal pressure profiles of individual paws during walking. *n* = 8.

In contrast to these contact-related abnormalities, no significant difference was observed in the step sequence regularity index among groups (Figure 6I). The preservation of this global coordination measure indicates that the SNI phenotype was dominated by reduced use of the ipsilateral hind paw rather than by a detectable loss of overall step sequence organization. Representative gait-cycle diagrams and contact-intensity traces were consistent with this interpretation: SNI shortened and weakened ipsilateral hind paw contact within otherwise recognizable four-limb stepping sequences, whereas either treatment partially restored the duration and magnitude of the affected paw signal (Figure A4A–D).

The parallel improvement produced by fludarabine and AMG487 extends their convergent effects from stimulus-evoked hypersensitivity and DRG neuronal hyperexcitability to dynamic use of the affected limb. Rather than a generalized reorganization of locomotor coordination being demonstrated, the temporal and spatial engagement of the ipsilateral hind paw during walking was preferentially improved by both interventions. Together, these results provide an additional functional layer of pharmacological evidence linking STAT1 associated and CXCR3-dependent signaling to the consequences of peripheral nerve injury.

## 4. Discussion

Traditional studies of neuropathic pain have primarily emphasized abnormal excitability of peripheral sensory neurons and its interaction with central sensitization; however, the mechanisms that sustain these pathological changes after peripheral nerve injury remain incompletely understood [2,16]. Against this background, it is further suggested by our findings that the DRG is not merely a structure containing sensory neuronal somata and mediating signal transmission but may also harbor an SGC-mediated neuronal regulatory pathway that contributes to the maintenance of chronic pain through modulation of the inflammatory signaling microenvironment surrounding sensory neurons [5]. Specifically, we identified a distinct IFN SGCs transcriptional population that emerged after peripheral nerve injury and was characterized by enhanced STAT1-related transcriptional activity and increased chemokine signaling. Further support for the relevant cellular pathway following peripheral nerve injury was provided by histological analyses: CXCL10 expression was increased in SGCs, accompanied by increased CXCR3 expression in nociceptive neurons, providing evidence for a potential signaling connection between SGCs and sensory neurons. Local pharmacological intervention targeting STAT1-related signaling or the CXCR3 receptor within the DRG alleviated pain-related hypersensitivity, reduced SNI-associated neuronal hyperexcitability, and improved the use of the injured hindlimb during movement, indicating that this signaling pathway also has functional relevance. These findings suggest that IFN SGCs may represent an important contributor to pathological remodeling within the DRG [33,34].

One important finding of this study is that the current understanding of SGC responses after nerve injury extends from generalized cellular changes to specific molecular states and their potential functional heterogeneity under pathological conditions. Previous studies have generally characterized SGC activation on the basis of increased GFAP expression, morphological alterations, enhanced gap-junction coupling, or broad inflammatory responses [20]. Although these indicators reflect the response of SGCs to nerve injury, they primarily describe the overall reactive state of these cells and limited information is provided about the molecular features and potential functions of distinct activated SGC subpopulations [35]. In contrast, the injury-associated SGC state identified in this study suggests that SGC activation is not a single, homogeneous cellular process [3] but may instead comprise multiple cell populations with distinct transcriptional characteristics and functional tendencies [35]. This concept provides a potential molecular framework for understanding the complexity of SGC responses reported in previous studies and suggests that analyses based solely on broadly expressed reactivity markers may be insufficient to explain SGC functions in different pathological contexts. Further studies integrating lineage tracing, spatial transcriptomics, and cell-type-specific interventions are needed to clarify the origins and dynamic transitions of these molecular states, as well as their causal relationships with changes in sensory neuronal function.

The anatomical positioning of SGCs gives them a unique capacity to regulate the local microenvironment of sensory neurons [36]. Sensory neuronal somata are closely surrounded by SGCs, allowing SGCs to participate in the maintenance of ionic homeostasis, metabolic support, and paracrine signaling around the neurons [37]. Under physiological conditions, maintenance of normal neuronal function and homeostasis is primarily contributed by this close interaction [35]. Following nerve injury, however, it is suggested by the STAT1-related transcriptional changes observed in SGCs that their local regulatory functions may be altered [20]. These changes may reflect a sustained bidirectional regulatory process between SGCs and sensory neurons [38]. On the one hand, the molecular environment surrounding sensory neurons may be altered by SGC-associated inflammatory signals; on the other hand, these local changes may be responded to by neurons through remodeling of the expression of relevant receptors [34]. Among the candidate signaling pathways identified in this study, CXCL10, a canonical IFN-inducible chemokine [39], may represent a secreted output of the STAT1-associated activation state and act on neighboring sensory neurons expressing CXCR3 [40]. Through this paracrine pathway, inflammatory SGCs may influence adjacent sensory neurons [41,42], thereby linking STAT1-related SGC activation to local pronociceptive signaling. Although chemokines have traditionally been regarded primarily as signals that regulate immune-cell recruitment, it is suggested by previous studies and the present findings suggest that they may exert broader effects in the nervous system and participate directly or indirectly in the regulation of neuronal excitability and pain sensitivity [41,42,43]. Notably, previous studies have focused mainly on CXCR3 in immune cells [44,45], whereas it is indicated by our results that CXCR3 is expressed under physiological conditions in a small subset of large-diameter sensory neurons and is upregulated in small-diameter neurons after SNI. Because small-diameter neurons include a substantial proportion of nociceptive afferents [46], their sensitivity to local CXCL10 signaling may be increased by this remodeling of the receptor expression profile. In parallel, CXCL10 immunoreactivity in SGCs was enhanced by SNI, and CXCR3 signals in sensory neurons were increased. Together with the spatial proximity of these signals, the single-cell transcriptomic findings, and the ability of CXCR3 intervention to alleviate pain hypersensitivity and neuronal hyperexcitability, these observations suggest that the CXCL10–CXCR3 axis may represent an important signaling component through which IFN SGCs promote neuronal sensitization.

Current treatments generally target widely expressed neuronal ion channels, central neurotransmitter systems, or broadly acting inflammatory processes [2,47]. Because these targets are distributed across multiple tissues and cell types, such treatments often provide only limited relief and may be associated with dose-limiting adverse effects [48]. In contrast, our findings suggest that local modulation of the inflammatory microenvironment within the DRG may provide a more anatomically precise therapeutic strategy for neuropathic pain [42,49]. As a key interface between peripheral sensory input and the central nervous system, the DRG possesses a relatively distinct local microenvironment and specialized vascular barrier properties, which may make it particularly amenable to peripheral administration and localized intervention [50]. Mechanistically, the inhibition of STAT1-related signaling may limit the formation or maintenance of the IFN SGCs’ state and reduce the production of chemokines such as CXCL10, whereas the blockade of CXCR3 may further suppress downstream signaling induced by chemokines in sensory neurons or other DRG cell populations [51]. Accordingly, a stratified intervention targeting both the upstream inflammatory SGC state and downstream chemokine receptor signaling may provide a theoretical basis and potential therapeutic direction for the precise regulation of the inflammatory microenvironment within the DRG.

This study also involved several limitations. First, fludarabine is not a STAT1-specific inhibitor, and its analgesic effects may result not only from inhibition of STAT1 signaling but also from the nonspecific modulation of local inflammatory responses or other immune processes [28,52]. Therefore, the observed behavioral improvement cannot be attributed exclusively to the suppression of STAT1 signaling in SGCs. In addition, we did not directly assess STAT1 pathway activity in vivo, and further validation using more selective pharmacological tools or cell-type-specific genetic strategies is required. Second, the single-cell transcriptomic data were obtained from a public dataset with a limited number of biological replicates and no information allowing samples to be traced to individual animals [53,54]. Consequently, animal-to-animal variation and the dependence structure among samples could not be adequately accounted for, and rigorous analyses using the individual animal as the statistical unit were not feasible. Findings that were not further validated by histological, molecular, or functional experiments should, therefore, be regarded primarily as exploratory or hypothesis-generating evidence, and their robustness and reproducibility require confirmation in independent datasets with clearly defined animal origins and sufficient biological replication. Third, functional experiments were conducted primarily in male mice. Although this design helped reduce interindividual variability in pain-related behavioral responses, it also limited our ability to assess sex differences. Because sex hormones, immune responses, and neuroimmune interactions may influence pain mechanisms, whether the present findings can be generalized to female animals remains to be determined [55,56]. Fourth, although the immunofluorescence results showed that CXCL10 was mainly localized to SGCs and that CXCR3 was predominantly distributed in small-diameter sensory neurons, spatial colocalization alone cannot establish the directionality of signal transmission or demonstrate functional coupling between the two. Thus, whether SGC-derived CXCL10 directly acts on CXCR3 in sensory neurons remains to be determined through cell-type-specific interventions and functional experiments. Finally, IFN signaling may engage noncanonical pathways beyond STAT1, and the present study did not examine the independent or cooperative contributions of these pathways to pain-related outcomes [57]. Therefore, although our findings support the involvement of IFN/STAT1-related signaling and the CXCL10–CXCR3 axis in pain, their precise causal relationships require further investigation.

## 5. Conclusions

In conclusion, this study identified an IFN SGC subpopulation that contributes to inflammatory remodeling in the DRG following peripheral nerve injury. The activation of STAT1 in these cells induces a chemokine response exemplified by CXCL10, which may promote sensory-neuron sensitization through CXCR3 signaling and contribute to neuropathic pain. Together, these findings highlight pathological SGC–neuron communication mediated by the STAT1–CXCL10–CXCR3 axis and suggest that local targeting of this pathway in the DRG may represent a potential therapeutic strategy for neuropathic pain [3].

## Figures and Tables

**Table 1:** Primer sequences used for qPCR.

Gene	Primer Sequence (5′–3′)
Cxcl10-F	CCAAGTGCTGCCGTCATTTTC
Cxcl10-R	GGCTCGCAGGGATGATTTCAA
Isg15-F	AGTGATGCTAGTGGTACAGAACT
Isg15-R	CAGTCTGCGTCAGAAAGACCT
Cxcr3-F	CCTCAATCCGCTGCTCTATG
Cxcr3-R	CAGGATGATTCTCTCCGTGAAG
Stat1-F	TCACAGTGGTTCGAGCTTCAG
Stat1-R	CGAGACATCATAGGCAGCGTG
Gapdh-F	AGGTCGGTGTGAACGGATTTG
Gapdh-R	GGGGTCGTTGATGGCAACA

## Data Availability

The RNA-sequencing data analyzed in this study were obtained from the publicly available Gene Expression Omnibus database under accession number GSE155622. No new RNA-sequencing datasets were generated in this study. The R scripts used for the single-cell RNA-seq analysis are publicly available on Figshare at https://doi.org/10.6084/m9.figshare.33307005.

## Abbreviations

The following abbreviations are used in this manuscript:

aCSF	Artificial cerebrospinal fluid
AMG	AMG487
BSA	Bovine serum albumin
cDNA	Complementary DNA
CGRP	Calcitonin gene-related peptide
CPWL	Cold paw withdrawal latency
DRG	Dorsal root ganglion
Flu	Fludarabine
GO BP	Gene ontology biological process
GS	Glutamine synthetase
IFN SGC	Interferon-responsive SGCs
ISGs	Interferon-stimulated genes
NF200	Neurofilament protein
PBS	Phosphate-buffered saline
PFA	Perfluoroalkoxy
PWMT	Paw withdrawal mechanical threshold
PWTL	Paw withdrawal thermal latency
qPCR	Quantitative real-time polymerase chain reaction
scRNA-seq	Single-cell RNA sequencing
SGCs	Satellite glial cells
SNI	Spared nerve injury
UMAP	Uniform manifold approximation and projection
